# Excessive diagnostic testing in acute kidney injury

**DOI:** 10.1186/s12882-016-0224-8

**Published:** 2016-01-15

**Authors:** David E. Leaf, Anand Srivastava, Xiaoxi Zeng, Gearoid M. McMahon, Heather E. Croy, Mallika L. Mendu, Allen Kachalia, Sushrut S. Waikar

**Affiliations:** Division of Renal Medicine, Brigham and Women’s Hospital, 75 Francis Street, Boston, MA 02115 USA; Department of Health Policy and Management, Harvard School of Public Health, Boston, MA 02115 USA

**Keywords:** Acute renal failure, AKI, Diagnostic yield

## Abstract

**Background:**

The patterns, performance characteristics, and yield of diagnostic tests ordered for the evaluation of acute kidney injury (AKI) have not been rigorously evaluated.

**Methods:**

We characterized the frequency of AKI diagnostic testing for urine, blood, radiology, and pathology tests in all adult inpatients who were admitted with or developed AKI (*N* = 4903 patients with 5731 AKI episodes) during a single calendar year. We assessed the frequency of abnormal test results overall and by AKI stage. We manually reviewed electronic medical records to evaluate the diagnostic yield of selected urine, blood, and radiology tests. Diagnostic yield of urine and blood tests was determined based on whether an abnormal test affected AKI diagnosis or management, whereas diagnostic yield of radiology tests was based on whether an abnormal test resulted in a procedural intervention. In sensitivity analyses we also evaluated appropriateness of testing using prespecified criteria.

**Results:**

Frequency of testing increased with higher AKI stage for nearly all diagnostic tests, whereas frequency of detecting an abnormal result increased for some, but not all, tests. Frequency of detecting an abnormal result was highly variable across tests, ranging from 0 % for anti-glomerular basement membrane testing to 71 % for urine protein testing. Many of the tests evaluated had low diagnostic yield. In particular, selected urine and blood tests were unlikely to impact AKI diagnosis or management, whereas radiology tests had greater clinical utility.

**Conclusions:**

In patients with AKI, many of the diagnostic tests performed, even when positive or abnormal, may have limited clinical utility.

## Background

Acute kidney injury (AKI) is an increasingly common complication among hospitalized patients [1, 2], and is associated with increased mortality [3, 4], development of chronic kidney disease [5, 6], and increased resource utilization [7–9]. The optimal diagnostic approach to AKI is not well established.

The traditional approach to the differential diagnosis of AKI emphasizes three broad categories: pre-renal AKI, which results from inadequate perfusion of the kidneys; post-renal AKI, which results from obstruction to the flow of urine; and intrinsic causes of AKI, which can be due to injury or dysfunction of the glomeruli, other blood vessels, tubules, or the interstitium. A large number of diagnostic tests are available for the investigation of AKI. Determining which test to order in which patient with AKI should be based on estimates of pre-test probability from the clinical context and an appreciation of the diagnostic performance characteristics of the various tests available.

Previous studies have examined the utility of some AKI diagnostic tests in isolation, such renal ultrasonography [10] and urine microscopy [11], but none have comprehensively assessed the patterns and clinical utility of a wide range of diagnostic tests in patients with AKI. We performed this study to identify the frequency with which various urine, blood, radiology, and pathology tests are ordered in patients with AKI, and to assess their diagnostic yield.

## Methods

### Study design

We conducted a retrospective cohort study among inpatients at Brigham and Women’s Hospital (BWH). All protocols were approved by the BWH Institutional Review Board.

### Study cohort

BWH is a 777-bed tertiary care academic medical center that provides care to an ethnically and socioeconomically diverse population. We analyzed all hospitalizations of adult patients (age ≥ 18) who were admitted in 2010 and developed AKI. While participants could only be considered to have a single episode of AKI per admission, AKI during subsequent admissions was also included and considered a separate AKI event. Thus, data were analyzed at the AKI-event level. We excluded patients with end-stage renal disease (ESRD) on maintenance hemo- or peritoneal dialysis and those who had undergone kidney transplantation.

### Data collection

We obtained data through the Research Patient Data Registry, a central clinical data warehouse maintained by Partners Healthcare System, of which BWH is a member. We obtained information on patient demographics (age, gender, race, comorbidities) and on patterns of diagnostic testing for AKI, including urine, blood, and radiology testing, as well as renal biopsy.

### Definition and staging of AKI

We defined and staged AKI according to the serum creatinine (SCr)-based criteria established by the Kidney Disease Improving Global Outcomes (KDIGO) Work Group [12]. Urine output data were not available. AKI was defined as an increase in SCr ≥0.3 mg/dl over any 48 h time period during hospitalization or ≥50 % increase in SCr over baseline known or presumed to have occurred within 7 days. For patients meeting criteria for AKI, we identified the AKI stage based on the ratio of maximum SCr during hospitalization divided by the baseline SCr, according to consensus criteria [12]. Baseline SCr was defined as the mean of all available outpatient SCr measurements obtained 7 to 365 days prior to admission, a validated approach described previously [13]. If no outpatient SCr values were available, the lowest SCr measurement during hospitalization was used as the baseline.

### AKI etiology

We manually reviewed the electronic medical record (EMR) of 100 randomly-selected patients and recorded the AKI etiology where it was known or suspected. In cases where AKI etiology was suspected to be multi-factorial, we recorded the two etiologies judged to be of primary importance.

### Diagnostic tests

The following urine, blood, radiology, and pathology tests were included as AKI diagnostic tests: urine tests – urinalysis, sediment, sodium, creatinine, urea, eosinophils, total protein, and protein electrophoresis (UPEP); blood tests – anti-neutrophil cytoplasmic antibody (ANCA), anti-glomerular basement membrane (anti-GBM) antibody, cryoglobulins, complement component 3 (C3), complement component 4 (C4), free light chains, and protein electrophoresis (SPEP); radiology tests – renal ultrasound and computerized tomography of the abdomen/pelvis (CTAP); pathology test – renal biopsy.

Urine and blood test results were determined to be normal or abnormal based on reference ranges provided by the BWH central laboratory. However, some tests such as urine sodium have normal physiologic variation and thus reference ranges are not applicable. These tests were therefore not categorized as normal or abnormal.

### Radiographic analysis

We manually reviewed all radiology reports. We considered CTAP tests as an AKI diagnostic test only if the indication for the test specifically mentioned AKI, acute renal failure, elevated SCr, decreased urine output, or concern for hydronephrosis. We categorized renal ultrasound and CTAP tests as abnormal if any abnormality was documented except simple cysts and non-obstructing stones and masses noted on prior imaging and found to be stable in size.

### Diagnostic yield of abnormal tests

We evaluated the diagnostic yield of one urine test (eosinophils), four blood tests (SPEP, free light chains, ANCA, and cryoglobulins), and two radiology tests (renal ultrasound and CTAP). To evaluate diagnostic yield of urine and blood tests, we manually reviewed the EMR of patients with abnormal test results to determine whether the test affected the patient’s AKI diagnosis or management during that hospitalization or within 30 days of discharge. For example, for patients with an abnormal SPEP or serum free light chain test result we determined from progress notes and discharge summaries whether they were subsequently diagnosed with a plasma cell dyscrasia likely to be contributing to their AKI (e.g., cast nephropathy) or whether the test affected their subsequent management (e.g., intravenous fluids, chemotherapy, or additional testing such as renal biopsy or bone marrow biopsy). Additional examples are provided in Table 1. Since radiology tests often reveal abnormal findings that appear chronic or otherwise have limited immediate clinical relevance, we assessed the diagnostic yield of radiology tests based on whether an abnormal test resulted in a subsequent procedural intervention. For renal ultrasonography, abnormalities other than hydronephrosis (e.g., atrophy, cortical thinning, and other chronic changes) were not included in this analysis, since interventions are not typically performed for these findings.

**Table 1:** Examples of abnormal test results affecting AKI diagnosis or management

Abnormal Test	Examples of Affecting AKI Diagnosis or Management
*Urine and Blood Tests*	
U Eos	• Subsequent diagnosis of acute interstitial nephritis • Renal biopsy to evaluate for interstitial nephritis • Withdrawal of offending medication for interstitial nephritis • Treatment with glucocorticoids
SPEP and SFLCs	• Subsequent diagnosis of multiple myeloma, Waldenstrom’s Macroglobulinemia, or other paraprotein-related disorder involving the kidneys • Additional testing such as renal biopsy, bone marrow biopsy, or skeletal survey • Treatment with intravenous fluids, glucocorticoids, or chemotherapy
ANCA	• Subsequent diagnosis of ANCA vasculitis involving the kidneys • Additional testing such as renal biopsy or skin biopsy • Treatment with glucocorticoids, cyclophosphamide, rituximab, or plasmapheresis
Cryo	• Subsequent diagnosis of cryoglobulinemia involving the kidneys • Additional testing such as renal biopsy • Treatment with glucocorticoids, cyclophosphamide, rituximab, or plasmapheresis
*Radiology Tests*	
Renal ultrasound and CTAP	• Placement of a foley catheter • Placement of a percutaneous nephrostomy tube • Ureteral stone removal • Ureteral stent placement

Abbreviations: *U Eos* urinary eosinophils, *SPEP* serum protein electrophoresis, *SFLCs* serum free light chains, *ANCA* anti-neutrophil cytoplasmic antibody, *Cryo* cryoglobulins, *CTAP* computerized tomography of the abdomen and pelvis

### Sensitivity analysis #1

Some diagnostic tests commonly ordered for the work-up of glomerulonephritis (ANCA, anti-GBM, and complement levels) and paraproteinemias (SPEP, UPEP, and serum free light chains) may also be ordered for purposes unrelated to AKI. We therefore manually reviewed the EMR of 50 randomly-selected patients who were tested for one of the above glomerulonephritis labs and 50 randomly-selected patients who were tested for one of the above paraproteinemia labs, and we determined the approximate frequency with which these tests were obtained for purposes related or unrelated to AKI. We classified each test into one of three categories: 1) obtained for AKI evaluation; 2) not obtained for AKI evaluation; 3) unable to determine whether ordered for AKI evaluation.

### Sensitivity analysis #2

To evaluate the appropriateness of testing for glomerulonephritis and paraproteinemias in AKI, we manually reviewed the EMR of the same 100 patients above. We developed criteria to evaluate appropriateness of testing (Table 2) and applied those criteria to each patient. Further, we evaluated whether appropriateness of testing differed based on patient location (floor vs. ICU), ordering team (primary team vs. renal consult), and AKI stage.

**Table 2:** Criteria for determining appropriateness of glomerulonephritis and paraprotein testing in AKI

Glomerulonephritis/vasculitis (≥1 major category to be considered appropriate)	Paraprotein disease (≥ 1 major category to be considered appropriate)
A. Hematuria and/or Proteinuria (any of the following): • RBCs on urine microscopy >25 cells/hpf ^a^ • RBC cast(s) on urine sediment evaluation • ≥ 3+ proteinuria on urine dipstick or ≥ 1 g by quantification B. Extra-renal manifestation (any of the following): • Cutaneous lesions (livedo reticularis or purpura) • Ear, nose, or throat manifestations such as sinusitis, epistaxis, or nasal ulcers • Pulmonary disease including hemoptysis or CXR showing patchy or diffuse opacities and with absence of an alternative etiology (e.g. pneumonia) • Arthritis C. Rapidly progressive AKI • Increase in serum Cr >100 % in less than 24 h, and absence of an alternative etiology D. Known history of glomerulonephritis or vasculitis	A. Proteinuria • ≥ 3+ on urine dipstick or ≥ 1 g by quantification B. Known history of a plasma cell dyscrasia (including any of the following): • Monoclonal gammopathy of undetermined significance • Smoldering myeloma • Multiple myeloma • Waldenstroms Macroglobulinemia • Serum free light chain ratio ≥ 100^b^ C. Hypercalcemia of unknown etiology • > 11 mg/dl^b^ D. Anemia of unknown etiology • Hemoglobin < 10 g/dl^b^ E. Bone lesions • One or more osteolytic lesions on skeletal radiography, CT, or PET-CT^b^

^a^Not including RBCs due to foley trauma. ^b^Adapted from [17]

### Sensitivity analysis #3

Evaluation of diagnostic yield (described above) focused on the utility of abnormal tests only. We therefore manually reviewed the EMR of the same 100 patients above to determine the diagnostic yield of normal test results. Specifically, we determined the number of normal or negative tests that affected the patient’s AKI diagnosis or management during that hospitalization or within 30 days of discharge. Examples of how a normal test result could have influenced a patient’s management include: continuing with supportive care (*e.g.* intravenous fluids for pre-renal azotemia); performing or deferring a renal biopsy; and starting or discontinuing immunosuppressive medications or plasmapheresis.

### Statistical methods

Most of the data shown are descriptive. Continuous data are shown as median (interquartile range [IQR]). Fisher’s exact test was used to compare the frequency of appropriate testing based on patient location, ordering team, and AKI stage. All tests were two-tailed, and *P* < 0.05 was considered significant.

## Results

### Study cohort

The study cohort included 4903 patients with 5731 episodes of AKI (70.8 % of which were stage 1, 17.1 % stage 2, and 12.1 % stage 3). The number of patients admitted 1, 2, or ≥ 3 times with AKI was 4323 (75 %), 471 (8 %), and 109 (2 %), respectively. Additional baseline characteristics are shown in Table 3.

**Table 3:** Baseline characteristics

Variable	All AKI Stages (*N*=5731)	Stage 1 (*N*=4058)	Stage 2 (*N*=981)	Stage 3 (*N*=692)
Age (yr) – median (IQR)	64 (52–75)	64 (53–75)	62 (52–72)	64 (52–74)
Female sex (%)	49.6	49.7	54.1	42.6
Race (%)				
White	83.0	82.9	84.3	81.9
Black	8.9	8.7	8.4	10.7
Hispanic	5.3	5.6	4.6	4.7
Other	2.7	2.8	2.7	2.7
Comorbidities (%)				
Malignancy	28.6	30.1	27.0	21.7
Hypertension	26.3	29.4	22.8	13.3
Diabetes Mellitus	18.6	20.1	16.0	13.6
Congestive Heart Failure	18.1	19.4	15.2	15.0
Chronic Pulmonary Disease	13.2	14.6	11.8	7.2
Chronic Kidney Disease	11.2	11.4	6.6	16.6
Chronic Liver Disease	4.5	3.9	4.8	7.7
Laboratory values on admission – median (IQR)				
Serum creatinine, mg/dL	1.0 (0.8-1.5)	1.0 (0.7-1.3)	1.1 (0.8-1.5)	1.9 (1.0-3.6)
Hemoglobin, g/dL	11.1 (9.7-12.7)	11.2 (9.9-12.7)	11.1 (9.7-12.8)	10.3 (8.9-11.8)
WBCs, 1000/μL	8.9 (6.3-12.8)	8.7 (6.3-12.4)	9.6 (6.5-14.1)	9.3 (5.9-14.3)
Albumin, g/dL	3.5 (3.0-3.9)	3.6 (3.1-4.0)	3.4 (2.9-3.9)	3.2 (2.8-3.7)

Stages 1, 2, and 3 refer to AKI severity according to the criteria established by the Kidney Disease Improving Global Outcomes (KDIGO) Work Group [12] as follows: stage 1, increase in serum creatinine 1.5 to 1.9 times baseline or absolute increase ≥0.3 mg/dl; stage 2, increase in serum creatinine 2.0 to 2.9 times baseline; stage 3, increase in serum creatinine ≥3.0 times baseline, or increase ≥0.5 mg/dl to an absolute level ≥4.0 mg/dl, or initiation of renal replacement therapy

The major etiologies of AKI determined in a random subset of 100 AKI episodes were ischemic acute tubular necrosis (24 %), pre-renal azotemia (21 %), nephrotoxic acute tubular necrosis (10 %), cardiorenal syndrome (8 %), glomerulonephritis (5 %), obstruction (3 %), and hepatorenal syndrome (2 %). AKI etiology was unknown in 22 % of episodes. Etiologies for the remaining 5 % included acute interstitial nephritis (AIN), rhabdomyolysis, and tumor lysis syndrome.

**Fig. 1:**
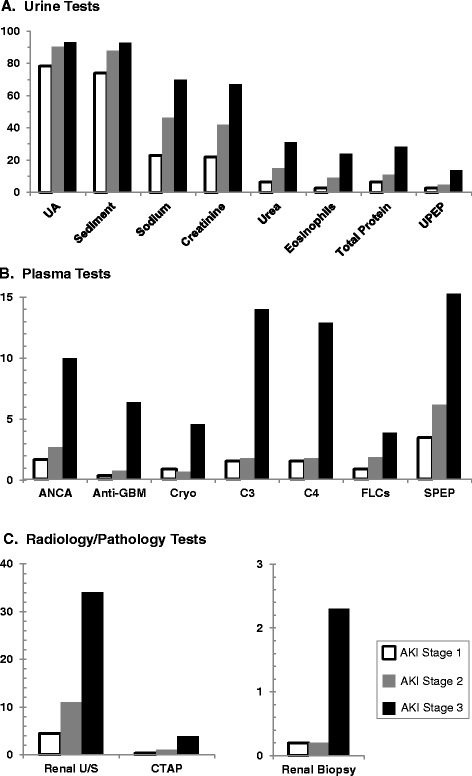
Frequency of diagnostic testing in AKI for **A**) urine tests, **B**) plasma tests, and **C**) radiology/pathology tests.. Frequency (%) is given for obtaining each diagnostic test at least once per episode of AKI. Abbreviations: UA, urinalysis; UPEP, urine protein electrophoresis; ANCA, anti-neutrophil cytoplasmic antibody; GBM, glomerular basement membrane; Cryo, cryoglobulins; C3, complement component 3; C4, complement component 4; FLCs, free light chains; SPEP, serum protein electrophoresis; CTAP, computerized tomography of the abdomen and pelvis

The most commonly ordered tests in AKI were urinalysis and automated urine sediment examination (Fig. 1). Blood tests for vasculitis, glomerulonephritis, and paraproteinemias were ordered in fewer than 10 % overall, and more frequently for higher stages of AKI. Radiology testing with renal ultrasonography or CTAP was obtained in 10 % and 1 %, respectively. Kidney biopsies were performed in 28 patients (0.5 % of all AKI episodes).

### Diagnostic yield of selected tests

**Fig. 2:**
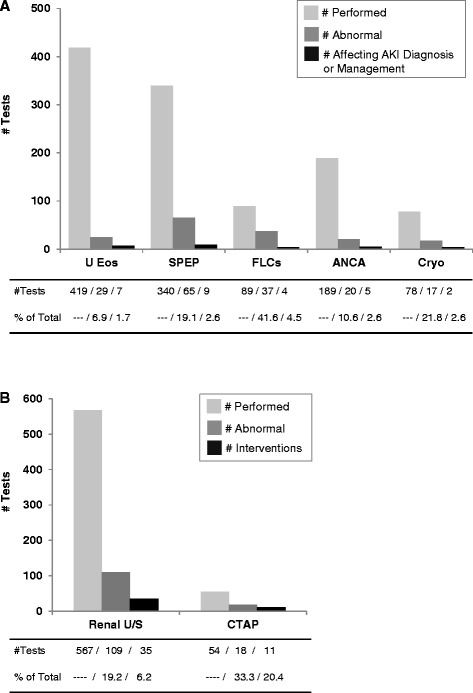
Diagnostic Yield. (**a**) Diagnostic yield was evaluated for blood and urine tests based on the number of abnormal tests that affected AKI diagnosis or management. (**b**) Diagnostic yield was evaluated for radiology tests based on the number of abnormal tests that resulted in a procedural intervention. For renal ultrasonography, abnormalities other than hydronephrosis (e.g. atrophy, cortical thinning, and other chronic changes) were not included in this analysis, since interventions are not typically performed for these findings. Abbreviations: U Eos, urinary eosinophils; SPEP, serum protein electrophoresis; FLCs, serum free light chains; ANCA, anti-neutrophil cytoplasmic antibody; Cryo, cryoglobulins; CTAP, computerized tomography of the abdomen and pelvis

Not all diagnostic tests during the course of AKI are obtained specifically for clarification of the cause or treatment of AKI (e.g., automated urinalysis, urine sodium, urine total protein). We therefore focused on those tests used most commonly to diagnose specific causes of AKI such as interstitial nephritis, paraprotein-related kidney disease, glomerulonephritides, and obstructive uropathy. Data on the number of tests performed, the number that were abnormal, and the number that affected AKI diagnosis or management are shown in Fig. 2. Additional data on frequency of abnormal results by AKI stage are shown in Table 4.

**Table 4:** Frequency of abnormal results

Test	Cutoff for “abnormal”	All Stages (*N* = 5731)	Stage 1 (*N* = 4058)	Stage 2 (*N* = 981)	Stage 3 (*N* = 692)
Urine Tests					
Eosinophils	≥1 %	6.9	5.8	7.2	7.5
Total Protein	>15 mg/dl	70.8	63.0	64.3	83.3
UPEP	M spike	7.9	5.0	9.8	10.0
Blood Tests					
ANCA	Titer > 1:20	10.6	8.0	3.7	14.9
Anti-GBM	≥1.0	0	0	0	0
Cryoglobulin	>0 %	21.8	26.3	0	21.2
C3	<90 mg/dl	36.5	26.9	21.1	45.6
C4	<10 mg/dl	12.9	11.6	10.5	14.3
Free Light Chains	See below	41.6	44.7	42.9	36.7
SPEP	M spike	19.1	16.4	23.9	19.7
Radiology Tests					
Renal Ultrasound	See Methods	34.6	40.6	23.4	34.9
CT Abdomen/Pelvis	See Methods	33.3	26.7	27.3	39.3

Frequency (%) of abnormal results was calculated by dividing the #tests ordered by the #abnormal results, multiplied by 100. Urinary tests with normal physiologic variation (e.g. sodium) were not included since there is no defined cutoff for “abnormal.” Similarly, renal biopsy was not included. Serum free light chains were considered abnormal if the Kappa/Lambda ratio was <0.26 or >1.65. Abbreviations: UPEP, urine protein electrophoresis; ANCA, anti-neutrophil cytoplasmic antibody; GBM, glomerular basement membrane; C3, complement component 3; C4, complement component 4; SPEP, serum protein electrophoresis

#### Urine eosinophils

Urine eosinophil testing was obtained in 7 % of AKI episodes (3 %, 9 %, and 24 % in patients with AKI stages 1, 2, and 3, respectively). Abnormal results (urine eosinophils ≥ 1 %) were found in 7 % of tests. Among the abnormal results, 24 % were in stage 1, 24 % were in stage 2, and 52 % were in stage 3. The median urine eosinophil % among those with abnormal results was 2 (IQR, 1-3). Among those with abnormal results, the presumptive diagnosis was AIN in 28 %. Kidney biopsy was performed in two patients with eosinophiluria and showed AIN in one, and vasculitis in the other.

#### SPEP

SPEP was ordered in 6 % of all AKI episodes and was abnormal in 19 %. Among AKI episodes with an abnormal SPEP, 75 % were IgG, 12 % were IgA, 8 % were IgM, and in 5 % only faint free light chains were identified; overall, 63 % consisted of monoclonal kappa and 37 % consisted of monoclonal lambda; the median M-spike concentration was 0.9 (IQR, 0.5-2.4 g/dl). In only 9 cases (2.6 % of the tests) did a positive SPEP affect AKI diagnosis or management (Fig. 2).

#### ANCA and anti-GBM

ANCA testing was ordered in 3 % of AKI episodes. Abnormal results were found in 11 % of tests, with median titers of 1:20 (IQR, 1:20 to 1:180). Among the abnormal results, 80 % were positive for p-ANCA and 20 % for c-ANCA. In only 5 cases (3 % of the tests) did a positive ANCA affect diagnosis or management (Fig. 2). In 3 of those cases, pauci-immune crescentic glomerulonephritis was confirmed by renal biopsy; in the remaining 2 cases, a presumptive diagnosis of ANCA vasculitis was made clinically. Anti-GBM antibodies were tested in 1 % of AKI episodes and were negative in all.

#### Radiology testing

Renal ultrasound was ordered in 567 (10 %) of AKI episodes. Abnormal results were detected in 196 (35 %) of the tests. Severe abnormalities such as severe or bilateral hydronephrosis were less frequently found (2 % and 10 %, respectively; Table 5). Hydronephrosis was detected in 109 (19 %) of the tests, among which 35 (32 %) resulted in a procedural intervention (Fig. 2). Among the 35 cases of that resulted in an intervention, a clinical history of metastatic or genitourinary cancer (*N* = 26), hemorrhagic cystitis (*N* = 2), and other comorbidities raising suspicion for obstruction were present in each case.

**Table 5:** Renal ultrasound abnormalities

	All Stages (*N* = 567)	Stage 1 (*N* = 187)	Stage 2 (*N* = 111)	Stage 3 (*N* = 269)
Any abnormality – no. (%)	196 (35)	76 (41)	26 (23)	94 (35)
Hydronephrosis – no. (%)	109 (19)	37 (20)	20 (18)	52 (19)
Mild – no. (%)	63 (11)	24 (13)	12 (11)	27 (10)
Moderate – no. (%)	33 (6)	10 (5)	5 (5)	18 (7)
Severe – no. (%)	13 (2)	3 (2)	3 (3)	7 (3)
Unilateral – no. (%)	52 (9)	24 (13)	11 (10)	17 (6)
Bilateral – no. (%)	57 (10)	13 (7)	9 (8)	35 (13)
Increased echogenicity – no. (%)	70 (12)	28 (15)	5 (5)	37 (18)
Cortical thinning – no. (%)	27 (5)	18 (10)	3 (3)	6 (2)
Atrophy – no. (%)	10 (2)	5 (3)	1 (1)	4 (1)

CTAP testing was ordered in 0.9 % of AKI episodes. Abnormal results were detected in 18 out of 54 (33 %) tests. Among the abnormal tests, 61 % resulted in an intervention (Fig. 2). CTAP, but not renal ultrasonography, was more likely to be abnormal among patients with higher AKI stages (Table 4). In general, the frequency of detecting abnormalities was greater with radiology tests than with urine or blood tests.

#### Renal biopsy

Renal biopsies were obtained in 28 patients (AKI stage 1 [*N* = 10], stage 2 [*N* = 2], and stage 3 [*N* = 16]). The diagnoses were immune-complex glomerulonephritis (22 %), diabetic kidney disease (14 %), pauci-immune crescentic glomerulonephritis (14 %), AIN (14 %), acute tubular injury (7 %), focal segmental glomerulosclerosis (7 %), and other miscellaneous diagnoses of lower incidence (*N* = 1 each, or 3.6 %): thrombotic microangiopathy, minimal change disease, membranous glomerulopathy, lupus nephritis, advanced chronic changes, and chronic tubulointerstitial nephritis.

### Sensitivity analyses

We conducted the following sensitivity analyses among 50 randomly selected patients who were tested for one of the “glomerulonephritis” labs (ANCA, anti-GBM, or complements), and an additional 50 randomly selected patients who were tested for one of the “paraproteinemia” labs (SPEP, UPEP, and serum free light chains):

### Sensitivity analysis #1 – frequency of testing related or unrelated to AKI

Among the patients who were tested for one of the glomerulonephritis labs, 46 % were ordered specifically for AKI, 12 % were ordered for reasons unrelated to AKI, and 42 % were ordered for reasons which were not clearly documented. Among the 50 patients who were tested for one of the paraproteinemia labs, 44 % were ordered specifically for AKI, 6 % were ordered for reasons unrelated to AKI, and 50 % were ordered for reasons which were not clearly documented.

### Sensitivity analysis #2 – appropriateness of testing

**Fig. 3:**
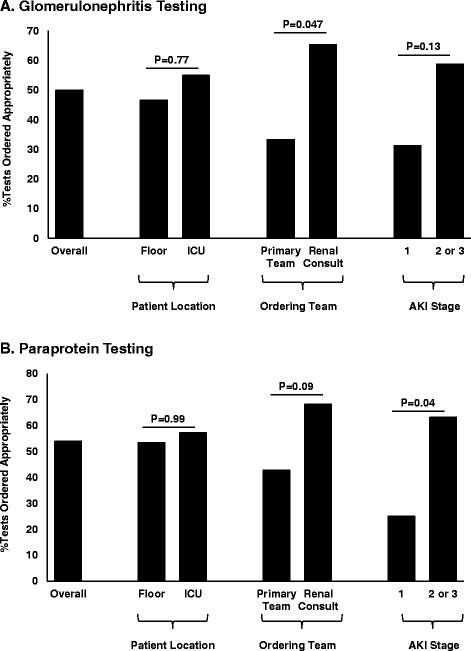
Appropriateness of diagnostic testing in AKI for **A**) glomerulonephritis and **B**) paraprotein testing

Using prespecified criteria (Table 2), we determined appropriateness of glomerulonephritis and paraproteinemia testing in AKI. Overall, glomerulonephritis and paraproteinemia testing was appropriate in 50 % and 54 % of tests, respectively (Fig. 3). When testing was ordered by the renal consult team compared to the primary team, appropriateness of testing was higher for glomerulonephritis testing (P=0.047), and showed a similar trend that did not reach statistical significance for paraproteinemia testing (P=0.09, Fig. 3). Similarly, when testing was ordered in patients with higher AKI stages, we found that appropriateness of testing was higher for paraproteinemia testing (P=0.04) but not for glomerulonephritis testing (P=0.13, Fig. 3).

### Sensitivity analysis #3 – diagnostic yield of normal tests

Among the patients who underwent one of the glomerulonephritis tests, testing was normal in 45 out of 50 (90 %) tests, and affected AKI diagnosis or management in only 1 out of the 45 (2.2 %) normal tests (and in 0 out of the 5 abnormal tests). Specifically, in one case a negative ANCA test resulted in discontinuation of immunosuppressive medications. Among the patients who underwent one of the paraproteinemia tests, testing was normal in 39 out of 50 (78 %) tests, and affected AKI diagnosis or management in 0 out of the 39 normal tests (and in 4 out of the 11 abnormal tests).

## Discussion

In this retrospective study we characterized the patterns and clinical utility of a wide variety of AKI diagnostic tests, including urine, blood, radiology, and pathology tests. Our key findings are: 1) frequency of testing increases with higher AKI stage for nearly all diagnostic tests; 2) frequency of abnormal results increases with higher AKI stage for some, but not all, diagnostic tests; and 3) many of the tests evaluated have low clinical utility. In particular, selected urine and blood tests are unlikely to impact AKI diagnosis or management. Cumulatively, these data suggest a paucity of actionable diagnostic tests in AKI.

Previous studies have examined the utility of a limited number of AKI diagnostic tests in isolation, including urine microscopy [11], urine eosinophils [14], the fractional excretion of sodium [15] and urea [16], and renal ultrasonography [10]. Most of these studies have focused on a single diagnostic test, have been limited by modest sample sizes, and have focused on test performance without inclusion of data on frequency of testing. None, to our knowledge, have simultaneously evaluated the patterns and test performance of a wide variety of AKI diagnostic tests in a large AKI cohort.

To assess diagnostic yield, we focused mostly on abnormal test results. Although normal test results can also be clinically useful by ruling out diagnoses, our sensitivity analysis of normal test results for glomerulonephritis and paraproteinemia labs revealed very low diagnostic yield. We therefore focused on abnormal test results, and evaluated diagnostic yield based on whether the result influenced AKI diagnosis or management. We found selected tests (*e.g.* urine eosinophils) are unlikely to be clinically useful in AKI. In contrast, renal ultrasonography and CTAP were much more likely to be useful, especially when the clinical history was suggestive. This was best exemplified by renal ultrasonography: among the cases of hydronephrosis that were detected and intervened upon, 100 % had a clinical history raising suspicion for obstruction. Thus, diagnostic testing should be based on both pre-test probability from the clinical context as well as an appreciation of the diagnostic value and actionability of the data provided by the test.

Among the urine, blood, and radiology tests examined in Fig. 2, the ratio of the number of tests ordered to the number of tests that had clinical utility – a concept similar to number needed to screen (NNS) – ranged substantially: from 5 in the case of CTAP to 60 in the case of urine eosinophils. In general the ratio was much higher for urine and blood tests, suggesting lower diagnostic yield, compared to radiology tests. In the case of renal ultrasonography, for example, the 567 tests ordered led to 35 interventions, resulting in a NNS of 16. This is similar to the NNS observed among high risk patients in a prior study that evaluated the utility of renal ultrasonography in AKI [10]. For urine and blood tests, on the other hand, we would speculate that clinicians may be aware of their poor predictive value, and therefore base most of their diagnostic and management decisions on other data such as clinical history and physical examination. For example, a recent study evaluated the utility of urine eosinophils in the diagnosis of AIN, using renal biopsies as the gold standard, and found that the presence of urine eosinophils performed very poorly in distinguishing AIN from other etiologies of AKI [14]. Abnormal radiology tests, on the other hand, may provide more actionable data, such as amelioration of obstruction.

We acknowledge several limitations. Some test results may have been considered abnormal based on reference ranges but may have been clinically insignificant. Other tests may have been ordered for reasons unrelated to AKI, such as routine urinalysis on admission, which may have led to overestimation of the frequency of AKI diagnostic testing. We attempted to minimize this bias by focusing on tests which are most commonly ordered for the diagnostic evaluation of AKI and not for other purposes. In the case of CTAP, testing was only included if ordered specifically for AKI. We also performed sensitivity analyses on tests for glomerulonephritis and paraproteinemias to determine the fraction of tests that were ordered for reasons unrelated to AKI. We found that only a minority of tests were ordered for reasons unrelated to AKI, though a large portion were also ordered for reasons which were not clearly documented.

Due to institutional restrictions we were unable to show data on the cost of diagnostic testing. An additional limitation is that because this was a single-center study the findings may not be generalizable to other settings; however, this limitation may be at least partially offset by the large sample size and heterogeneous nature of our patient population. Finally, we were unable to evaluate the diagnostic yield of AKI testing in selected subgroups, such as patient location (general medical floor versus ICU), ordering team (primary team versus renal consult), or AKI severity. However, we conducted sensitivity analyses to evaluate whether appropriateness of diagnostic testing varied based on these factors. Although we found that appropriateness of testing may increase for some tests when ordered by the renal consult rather than the primary team, patients who require renal consultation may have greater severity of AKI; thus, we cannot exclude the possibility of confounding, and therefore this finding should be interpreted with caution.

## Conclusions

In summary, a large number of diagnostic tests are available for the evaluation of AKI, and determining which test to order should be based on estimates of pre-test probability and performance characteristics of the test. Our data suggest that many of the currently available tests have limited clinical utility, even when they are abnormal or “positive”. Thus, developing better diagnostic tests that provide reliable and actionable data on AKI diagnosis or management should be a priority in AKI research. The dearth of such tests may be a key reason why therapeutic advances to improve outcomes in AKI have been largely unsuccessful.

## Abbreviations

AKI: Acute kidney injury
ANCA: Anti-neutrophil cytoplasmic antibody
BWH: Brigham and Women's Hospital
CTAP: Computerized tomography of the abdomen/pelvis EMR Electronic medical record
EMR: Electronic medical record
ESRD: End-stage renal disease
GBM: Glomerular basement membrane
IQR: Interquartile range
KDIGO: Kidney disease improving global outcomes
NNS: Number needed to screen
SPEP: Serum protein electrophoresis
UPEP: Urine protein electrophoresis
